# Molecular damage and responses of oral keratinocyte to hydrogen peroxide

**DOI:** 10.1186/s12903-018-0694-0

**Published:** 2019-01-11

**Authors:** Kuan-Yu Lin, Ching-Hung Chung, Jheng-Sian Ciou, Pei-Fang Su, Pei-Wen Wang, Dar-Bin Shieh, Tzu-Chueh Wang

**Affiliations:** 1 Department of Biochemistry and Molecular Biology, Pennsylvania State University, State College, Harrisburg, PA 16803 USA; 2 Department of Stomatology, National Cheng-Kung University Hospital, Tainan, 70101 Taiwan; 3 Graduate Institute of Pharmaceutical Science, Chia-Nan University of Pharmacy and Science, Tainan, 71710 Taiwan; 4 Department of Statistics, National Cheng Kung University, Tainan, 70101 Taiwan; 5 Institute of Oral Medicine and Department of Stomatology, College of Medicine, National Cheng Kung University Hospital, National Cheng Kung University, Tainan, 70101 Taiwan; 6 Center of Applied Nanomedicine, Center for Micro/Nano Science and Technology, Advanced Optronic Technology Center, Innovation Center for Advanced Medical Device Technology, National Cheng Kung University, Tainan, 70101 Taiwan; 7 Department of Pharmacy, Chia-Nan University of Pharmacy and Science, Tainan, 71710 Taiwan

**Keywords:** Tooth bleaching, Molecular genetics, Oxidative stress, Toxicology, Keratinocyte(s)

## Abstract

**Background:**

Hydrogen peroxide (H_2_O_2_)-based tooth bleaching reagents have recently increased in popularity and controversy. H_2_O_2_ gel (3%) is used in a Nightguard for vital bleaching; transient tooth sensitivity and oral mucosa irritation have been reported. Genotoxicity and carcinogenicity have also been significant concerns.

**Methods:**

We used primary cultured normal human oral keratinocytes (NHOKs) as an in vitro model to investigate the pathological effects to mitochondria functions on human oral keratinocytes exposed to different doses of H_2_O_2_ for different durations.

**Results:**

An MTT assay showed compromised cell viability at a dose over 5 mM. The treatments induced nuclear DNA damage, measured using a single-cell gel electrophoresis assay. A real-time quantitative polymerase chain reaction showed H_2_O_2_ induced significant increase in mitochondrial 4977-bp deletion. Mitochondrial membrane potential and apoptosis assays suggested that oxidative damage defense mechanisms were activated after prolonged exposure to H_2_O_2_. Reduced intracellular glutathione was an effective defense against oxidative damage from 5 mM of H_2_O_2_.

**Conclusion:**

Our study suggests the importance for keratinocyte damage of the dose and the duration of the exposure to H_2_O_2_ in at-home-bleaching. A treatment dose ≥100 mM directly causes severe cytotoxicity with as little as 15 min of exposure.

## Background

Tooth color contributes significantly to a person’s aesthetic appearance. Normal tooth color is determined by the optical and chromatic properties of dentine and enamel: hue, value, chroma, thickness, texture, and translucency [1]. Both intrinsic and extrinsic factors in dentin or enamel, or in both, can cause tooth discoloration [1–3]. Nightguard vital bleaching (at-home-bleaching) was first introduced in 1989 to reduce tooth discolorations [4]. The 10% carbamide peroxide used in a custom-fitted nightguard was converted by saliva in the oral cavity to 3% H_2_O_2_ (~ 0.9 M) and 7% urea [3–5]. H_2_O_2_ diffuses into the enamel-dentine junction and dentine, and using a redox reaction, decomposes chromogens, which whitens teeth [2, 6]. The most noteworthy side effects associated with at-home-bleaching are tooth sensitivity and oral mucosa irritation. Some studies [5, 7, 8], however, report that these side effects are transient and can be recovered from after treatment. Safety for exposure to a long-term H_2_O_2_ bleaching agent has also been a concern in more recent studies [9, 10]. H_2_O_2_ generates free radicals, especially hydroxyl radical, in oxidative reactions; free radicals are considered genotoxic and carcinogenic [11, 12]. Long-term exposure to high-dose H_2_O_2_ can damage soft and hard oral tissue [4, 6]. The inappropriate application or abuse of H_2_O_2_ during cleaning treatments has other potential adverse effects [13].

We explored the pathological effects of H_2_O_2_ on gingival mucosa under different concentration doses and exposure durations using primary cultured normal human oral keratinocytes (NHOKs) as a model. Gingival mucosa is composed of highly-regenerative keratinized stratified squamous epithelium and submucosal connective tissues. The NHOKs in the epithelium form a barrier to defend basal cells with regenerative capability from chemical and physical insults of oral environment including the reactive oxygen species generated during tooth bleaching [14]. Through previous oral transmucosal bioavailability studies, various factors were known to reduce the chemical exposure to basal cells thus reducing the actual H_2_O_2_ concentration at this level from tooth bleaching [15]. The keratinized gingival mucosa is known to harbor a lower permeability, intermediate residence time, and high blood flow as compared to the buccal and palatal muocosa [16, 17]. H_2_O_2_ gel also effectively reduced risk of high dose exposure to oral mucosa compared to the liquid form [18]. Besides, the EU Commission established in the cosmetics directive that the concentration of hydrogen peroxide in consumer oral hygiene products should be limited to 0.1% [19]. Therefore, we tested the proposed equivalent exposure dose at basal level in the range of 0.01~100 mM (0.000033% ~ 0.33%) in this study. The mechanism of H_2_O_2_-induced cell death was further explored by assessing mitochondrial membrane potential- and apoptosis-related pathways. The antioxidant defense system was also investigated for its role in the short- and the long-term exposure.

## Methods

### Normal human Oral keratinocytes: Primary cultures

Gingival tissue was isolated from excess tissue during the surgical removal of impacted wisdom teeth from 10 healthy patients (age 19~50) under approval by NCKUH Institutional Review Board for Human Studies. The specimens were incubated in 1 mL of Keratinocyte-SFM medium (KGM; Gibco BRL, Gaithersburg, MD, USA) containing dispase (5 mg/mL) (Gibco) at 4 °C for overnight. The epithelial tissue was separated from the specimens and washed with phosphate-buffered saline (PBS) containing penicillin, streptomycin, and fungizone (PSF 100X; Gibco) before the specimens were dissected into smaller pieces (about 1 mm^3^). The tube was centrifuged at 800 rpm for 5 min, and then the pellet was transferred to another tube containing 1 mL of 0.25% trypsin (Gibco) and incubated at 37 °C for 5–7 min. One milliliter of 0.25% trypsin inhibitor (Gibco) and KGM (1 mL) were subsequently added. The tube was centrifuged again at 1200 rpm for 5 min and the pellet was resuspended with KGM (5 mL) and then incubated in a T25 flask containing 100X lincomycin (10 mg/mL) (Sigma-Aldrich, St. Louis, MO, USA). The procedure was modified from Kim et al. [20], and the cells were plated with a density < 80% for the initiation of treatment. The pooled NHOKs samples from 10 donors were used within 3rd to 10th passages. The NHOKs were characterized periodically for microscopic presentation and their intermediate filament expression profile (keratin+, vimentin-) as well as free of mycoplasma contamination by qPCR. The results well defined the NHOKs in this study. The pooled NHOKs were used for all tests and the experiments were triplicated.

### Comet assay

NHOKs (2 × 10^5^/well) were seeded in 6-well plates overnight and then the old medium was replaced with new medium containing different doses of H_2_O_2_ for 1 h or 8 h. Comet assays (single cell gel electrophoresis [SCGE]) were performed according to the manufacturer’s instructions (Trevigen, Gaithersburg, MD, USA). Briefly, 10 μL of the resuspended solution was mixed with melted 75 μL of 1.5% LM agarose then 50 μL of the final mixture was immediately placed onto a CometSlide™. The slides were then placed at 4 °C in the dark for 15 min before immersed in 4 °C lysis solution for 1 h. The slides were then immersed in alkaline DNA unwinding solution (pH > 13.0) at 25 °C in the dark for 30 min and placed in an electrophoresis tray. Electrophoresis was performed (30 Volt 300 mA) for 20 min and the slides were then processed in neutralization buffer for 5 min for 3 times, then immersed in methanol for 5 min. The slides were dried at 37 °C for another 5 min then stained by 100 μL of diluted SYBR Green I (Ex = 450–490 nm; Em = 510 nm) for 30 min. After electrophoresis, levels of DNA damage were quantified by the presence of “comet tail” viewed under epifluorescence microscopy, where the migration of DNA toward the anode increased as the frequency of single strand break (SSB) increased. One hundred cells were randomly selected from each slide to calculate the DNA damage level with comet assay software. The damage levels were determined by tail moment (TM) and calculated by the following equations.

$$ \mathrm{Taillength}\left( TL,\%\right)=\left(\mathrm{contentextent}-\mathrm{headextent}\right)/\mathrm{cometextent}\times 100\% $$

$$ \mathrm{Tailintensity}\left( TI,\%\right)=\left(\mathrm{cometintegratedintensity}-\mathrm{headintensity}\right)/\mathrm{cometintegratedintensity}\times 100\% $$

$$ \mathrm{Tailmoment}(TM)=\mathrm{TL}\times \mathrm{TI} $$

### Total DNA isolation

NHOKs (5 × 10^5^/well) were seeded with different doses of H_2_O_2_ and incubated for 1 h or 8 h. The cells were then harvested and counted after they had been stained with 0.4% trypan blue dye (Sigma-Aldrich). They were then transferred to 1.5-mL microtubes, and DNA was isolated using Trizol (Sigma-Aldrich). The final precipitated DNA was dissolved in 100 μL of ddH_2_O and calculated for the concentration by spectrophotometer (2000c, NanoDrop, Wilmington, DE, USA) at 260/280 nm.

### mtDNA^4977^ deletion assay

The mitochondrial DNA copy number and the ratio of mtDNA^4977^ deletion to the total mtDNA were determined using a real-time quantitative polymerase chain reaction (qPCR) and Power Sybr Green (Applied Biosystems, Foster City, CA, USA) in a qPCR system (StepOne™; Applied Biosystems) as previously described [21]. The primers designed for quantification of total mtDNA were listed as follow: L11 5′-ATACAGACCAAGAGCCT-3′ (forward; nt5527–5543) and H11 5′-GCGGGAGAAGTAGATTGA-3′ (reverse; nt5722–5739). The mtDNA4977 deletion was determined by targeting the template spanning from nt8469 to nt13447 with the primers: L1 5′-AACCAACACCTCTTTACAGTGAA-3′ (forward; nt8342–8364) and H1 5′-GATGATGTGGTCTTTGGAGTAGAA-3′ (reverse; nt13501–13,524). ΔmtDNA4977 deletion data obtained from qPCR were calculated with the following equation. ΔmtDNA^4977^ deletion = mtDNA^4977^/total mtDNA.

### Quantitating reduced glutathione

NHOKs (2 × 10^5^/well) were treated with different doses of H_2_O_2_ for 1 h or 8 h and then harvested. The cells were incubated at 37 °C for 20 min with 500 μL of 1X 20-μM of CellTracker Green CMFDA Dye (Molecular Probes, Eugene, OR, USA) and then centrifuged at 1200 rpm (241 *g*) for another 5 min. They were then washed three times with 500 μL of PBS, resuspended, and transferred to flow tubes. The glutathione (GSH) content, determined using CMFDA (green, Ex = 522 nm; Em = 595 nm), was quantitated using a flow cytometer (FACSCalibur; BD Biosciences, Franklin Lakes, NJ, USA) at the FL1-H channel, as previously described [22].

### Apoptosis assay

H_2_O_2_-treated NHOKs (1 × 10^6^/mL) were harvested and resuspended in annexin-V binding buffer (BioVision, Milpitas, CA, USA). A 100-μL aliquot of the solution was transferred to a 5-mL culture tube, and 5 μL of annexin V-FITC (BD Biosciences) was added. The cells were gently mixed and then incubated for 15 min at room temperature in the dark. At the end of the inoculation period, 400 μL of annexin V binding buffer was added to each flow tube. Late and early apoptosis was evaluated using the flow cytometer and analyzed using WinMDI 2.9 software (The Scripps Research Institute; http://www.cyto.purdue.edu/flowcyt/software/Winmdi.htm) [23].

### Cell viability assay

An MTT (1-(4,5-dimethylthiazol-2-yl)-3,5-diphenylformazan) assay was used to measure cell viability, as previously described [24]. Briefly, the MTT reagents were added to the cultured cells subject to different concentrations and exposure period of H_2_O_2_ with a final concentration at 0.5 mg/mL. The cells were then incubated for 3 h at 37 °C. The medium was removed and DMSO was added into each well to dissolve the formazan. Finally, the cell viability was determined by the optical absorption at 490 nm referenced to the control.

### Mitochondrial membrane potential (ΔΨ_m_) analysis

JC-1 (5,5′,6,6′-tetrachloro-1,1′,3,3′-tetraethylbenzimidazolcarbocyanine iodide) (Molecular Probes) was used to quantify ΔΨ_m_ (monomers at a low ΔΨ_m_ presented a green fluorescent emission [green, Ex = 488 nm; Em = 527 nm] and aggregates formed at a high ΔΨ_m_ showed red fluorescence [red, Ex = 488 nm; Em = 590 nm]). NHOKs (2 × 10^5^ cells/well) were seeded in 6-well plates and incubated overnight before treated with different concentrations of H_2_O_2_ for either 1 h or 8 h. The test media were removed, then the cells were harvested and washed with PBS before adding 500 μL of 10 μM JC-1 was added to the media followed by gentle vortex. The cells were incubated at 37 °C for 20 min, then centrifuged at 1200 rpm (241 *g*) for 5 min, and then the pellet was washed 3 times in 500 μL of PBS. The cells were re-suspended in 500 μL of PBS and then transferred to a flow tube for FACS analysis (FACSCalibur; BD Biosciences, Franklin Lakes, NJ, USA). The ratio between red and green JC-1 fluorescence signals was calculated from data acquired in the flow cytometer at FL1-H and FL2-H, as previously described [24].

## Results

### Nuclear DNA damage and cell cytotoxicity were higher in cells treated with H_2_O_2_

**Fig. 1:**
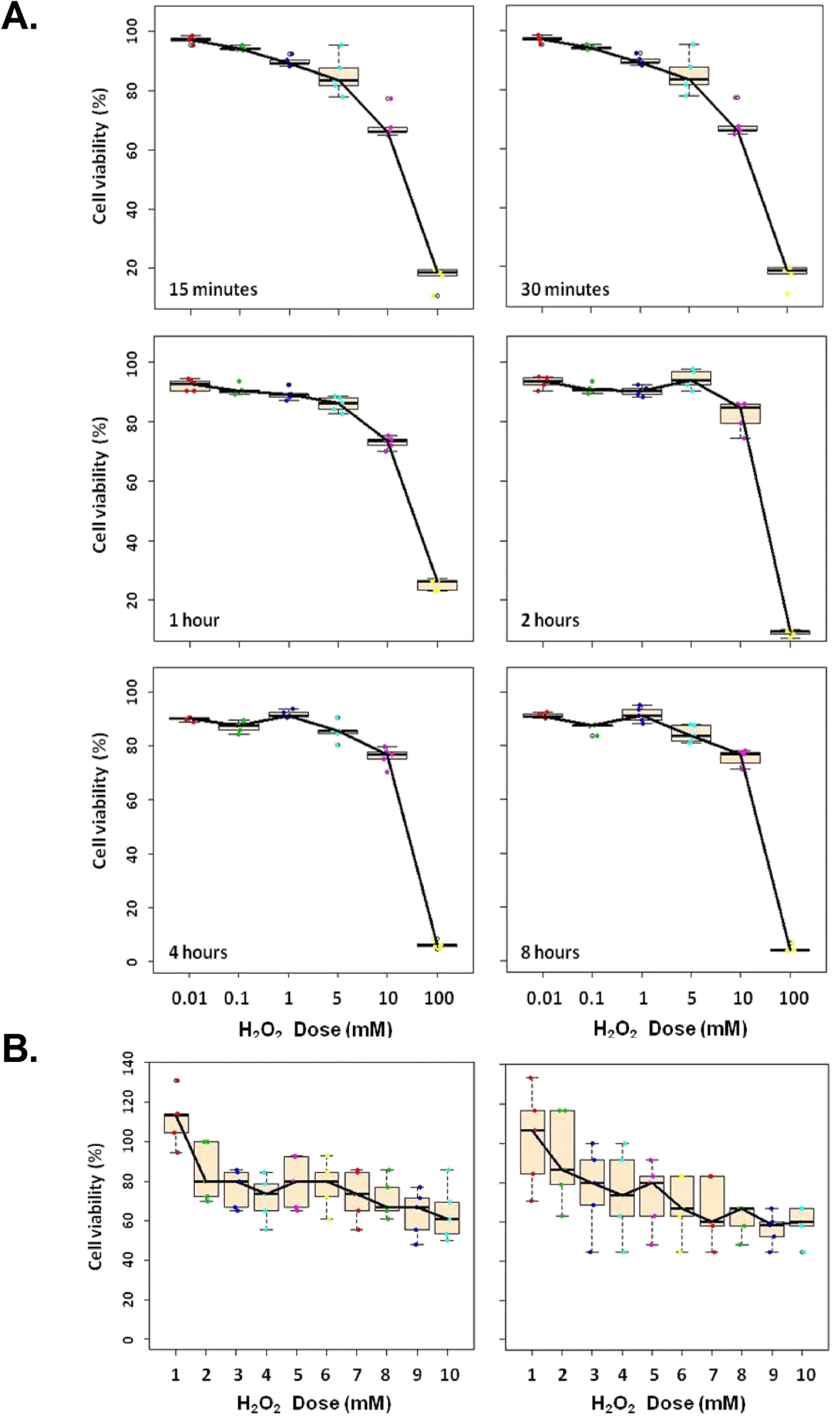
Cell viability of NHOKs after they had been exposed to different doses of H_2_O_2_ for different durations. (**a**) NHOKs were seeded in medium containing H_2_O_2_ (range: 0.01–100 mM). The cells were incubated for 15 min, 30 min, 1 h, 2 h, 4 h, and 8 h. Cell viability in culture medium alone was the negative control. The experiment was repeated 5 times. (**b**) Cell viability of NHOKs cultured in medium containing 10 different doses of H_2_O_2_ (range: 1–10 mM). The experiment was repeated 5 times

Cell viability based on the dose of H_2_O_2_ was analyzed by MTT assay, which showed that regardless of the incubation period (between 15 min and 8 h); the H_2_O_2_-treated NHOKs were > 90% viable when treated with 0.01–1 mM of H_2_O_2_. After the NHOKs had been treated with 5 mM of H_2_O_2_, however, their viability was significantly lower: about 85%. Moreover, cell viability was inversely dependent upon exposure time and dose: the longer and higher they respectively were, the lower was cell viability. Cells treated with > 5 mM of H_2_O_2_ were significantly less viable (Fig. 1). The critical dose for a significant and substantial reduction in cell viability for all exposure times was 5 mM (Fig. 1a). A more detailed dosage analysis (1–10 mM in 1-mM increments) showed that viability was dose-dependent as well as time-dependent (Fig. 1b). We chose 5 mM of H_2_O_2_ as the treatment dose.

**Fig. 2:**
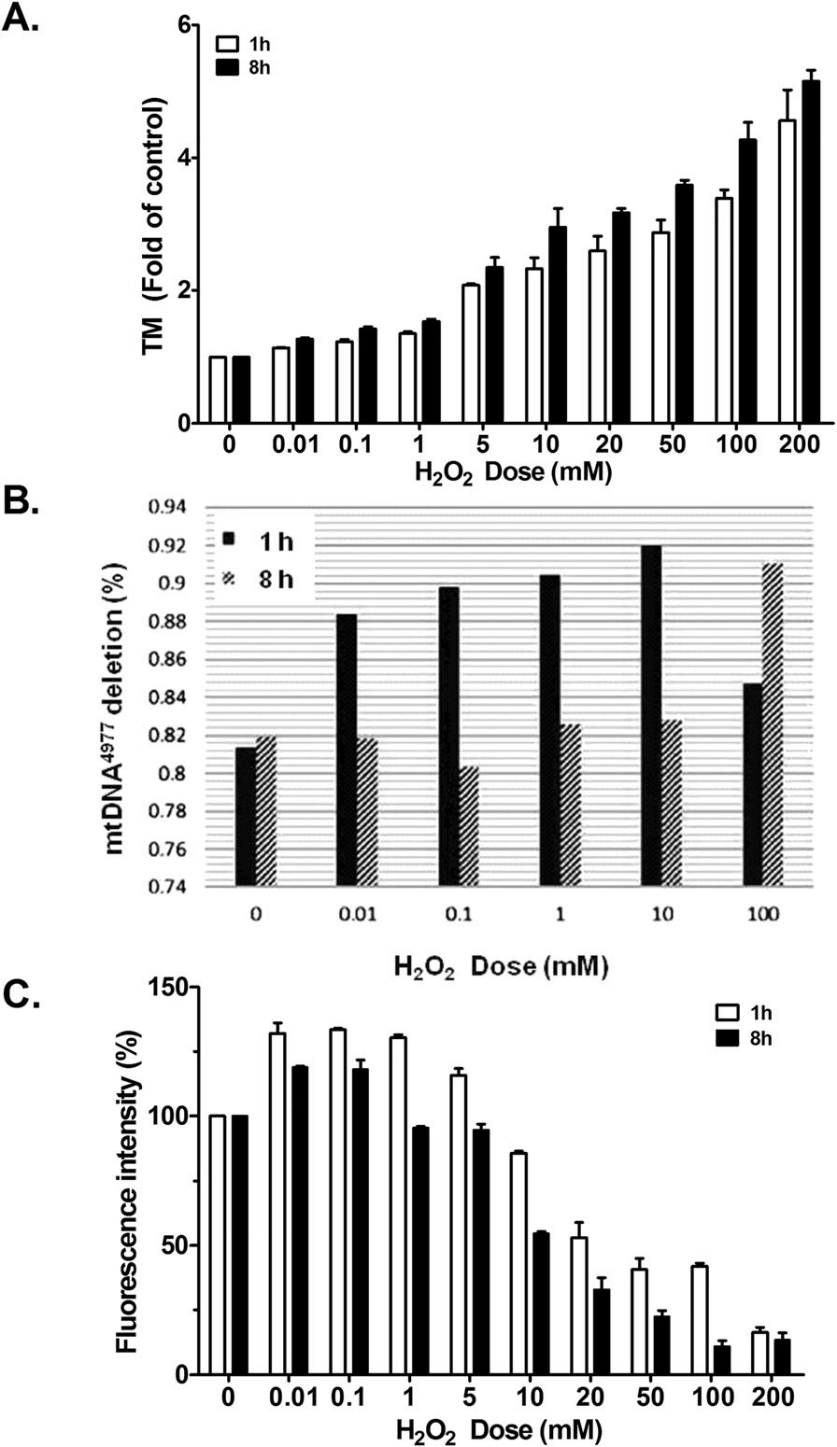
DNA damage and glutathione (GSH) levels increased after the NHOKs had been exposed to H_2_O_2_. (**a**) A comet assay was used to analyze the nDNA damage caused by H_2_O_2_-induced oxidative attacks on NHOK DNA. The cells were exposed to H_2_O_2_ for 1 h or 8 h. Data are shown in folds, by comparing the changes to the control at 0 mM (set as 1). The experiment was repeated 3 times, and the values are expressed as mean (± SD). (**b**) qPCR was used to measure mtDNA4977 deletion for each dose after NHOKs had been exposed to H_2_O_2_ for 1 h and 8 h. The experiment was repeated 4 times and the values are expressed as mean (± SD). (**c**) The quantity of intracellular GSH was determined by measuring the CMF that remained in the NHOKs. Data are expressed as a % of fluorescence intensity. The untreated cell culture was the negative control (set as 100%). The experiment was repeated 3 times, and values are expressed as mean (± SD)

An alkaline comet assay was used to observe the nuclear DNA (nDNA) damage caused by H_2_O_2_-induced oxidative attacks. The tail moment (the product of the tail length and the fraction of total DNA in the tail) was significantly H_2_O_2_-dependent (Fig. 2a). The tail moment increased substantially but nonsignificantly: it doubled (and was otherwise dose-dependent except at 100 mM) after the NHOKs had been exposed to 5 mM of H_2_O_2_ for 1 h.

### Mitochondrial DNA (mtDNA) deletion and glutathione inhibition increased after the NHOKs had been briefly exposed to H_2_O_2_

The in vitro mtDNA^4977^ deletion assay showed that NHOKs contained an average of 0.81% of the baseline ratio. After 1 h of treatment with 0.01–10 mM of H_2_O_2_, mtDNA^4977^ deletion was dose-dependently higher, but after treatment with 10 mM of H_2_O_2_, mtDNA^4977^ deletion was back at 85% of the baseline level. However, mtDNA^4977^ deletion did not significantly change in NHOKs treated with a dose of H_2_O_2_ < 10 mM for 8 h. Only after the NHOKs had been treated with 100 mM H_2_O_2_ for 8 h did mtDNA^4977^ deletion significantly increase to 0.91% (Fig. 2b).

The glutathione (GSH): oxidized glutathione (GSSG) ratio was inversely related to the proportion of mtDNA^4977^ deletion. GSH levels were significantly higher after NHOKs had been exposed to H_2_O_2_ (0.01 and 5 mM) for 1 h. However, GSH was significantly and dose-dependently lower after they had been exposed to doses > 10 mM. In 8 h of treatment, excess GSH was generated after treatment with 0.01 and 0.1 mM of H_2_O_2_, but when treatment doses were > 1 mM, GSH levels were lower (Fig. 2c). Not only was there a dose-dependent effect, but GSH levels were also time-dependently lower at all doses.

### The effect of H_2_O_2_ treatment on NHOK apoptosis activation via the loss of mitochondrial membrane potential

**Fig. 3:**
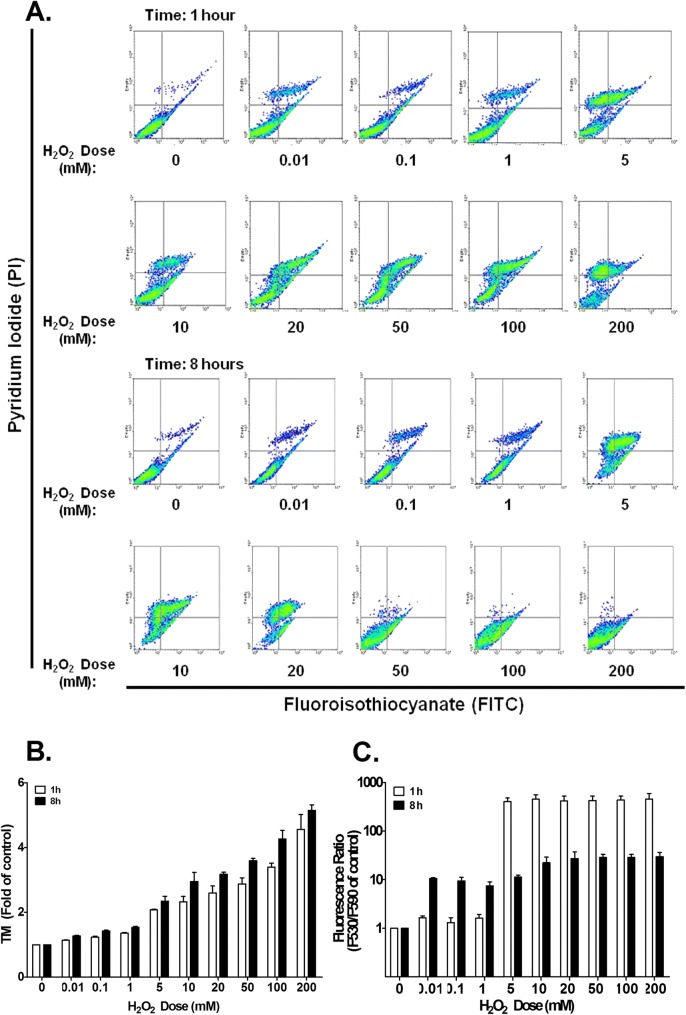
NHOK apoptosis activation by H_2_O_2_ treatment through mitochondrial membrane potential (ΔΨm) loss. (**a**) Annexin V-FITC/PI for 1 h and 8 h. (**b**) % apoptosis was calculated from the results in Fig. 3A. The Controls showed normal levels of apoptosis. The experiment was repeated 3 times, and the values are expressed as mean (± SD). (**c**) JC-1 was used to measure ΔΨm by comparing the amounts of green (F530) and orange fluorescence (F590). The fluorescence ratios (F530/F590) were then compared with that of the Controls (set as 1) and expressed in folds, to provide a clear view of the ΔΨm after exposing the NHOKs to H_2_O_2_ for either 1 h or 8 h. The experiment was repeated 3 times, and the values are expressed as mean (± SD)

Flow cytometric analysis showed NHOKs undergoing apoptosis in the annexin-V FITC-positive/PI (pyridium iodide)-negative population, and undergoing necrosis in the annexin-V FITC-negative/PI-positive population. After the NHOKs had been exposed to H_2_O_2_ for 1 h, there was a dose-dependent increase in the level of apoptotic cells, which began to fall when the dose was > 100 mM. In the 8-h treatment group, at doses < 10 mM, apoptosis was dose-dependently higher, but it gradually decreased when doses were > 20 mM (Fig. 3a, b).

We also analyzed the cellular apoptosis pathway with JC-1 staining as a marker for mitochondrial membrane potential (ΔΨ_m_). The loss of ΔΨ_m_ is an early event in intrinsic mitochondrial-mediated apoptosis. The FL1:FL2 fluorescence ratio reflects the status of ΔΨ_m_: a higher value indicates that more ΔΨ_m_ has been lost. Within 1 h, the ΔΨ_m_ levels returned to normal when H_2_O_2_ doses were < 1 mM. A significant amount of ΔΨ_m_ was lost, which was indicated by the 407-fold rise in the fluorescence ratio (Fig. 3c) when the treatment dose was ≥5 mM. However, the lost ΔΨ_m_ was partially restored at 8 h to 27-fold that of the controls. An increase in lost ΔΨ_m_ was significant at 8 h when treatment doses were between 0.01 and 5 mM, which was about 10-fold that of the negative.

## Discussion

After we exposed NHOKs to H_2_O_2_, we comprehensively evaluated the effects on cell viability, DNA damage, cellular defense response to oxidative damage, and apoptosis in the estimated basal cell level exposure dose range of 0.01~100 mM, which is about 1/10 that of the H_2_O_2_ in the bleaching gel. From the dose- and duration-dependent decrease in cell viability, we confirmed that exposing NHOKs to H_2_O_2_ induced significant cellular damage, especially when the dose exceeded 5 mM. The study also revealed that the degree of cell proliferation inhibition was dose- and duration-dependent. This finding is consistent with O’Toole et al., who used a similar dose range: 2, 4, and 7 mM [25]. They reported that a low dose of H_2_O_2_ have no effect on NHOKs viability at doses ≤700 μM for even after exposure for 24 h. We found that > 90% cell survival could be maintained using doses < 1 mM, even after 8 h of exposure, which is the exposure time to Nightguard vital bleaching.

Dental bleaching is based on the ability of H_2_O_2_ to penetrate through tooth structure and produce free radicals to oxidize the colored organic molecules. There are many reports investigated the effect of dose and exposure time of H_2_O_2_ to the pulpal tissues as the bleaching agents penetrated through the tooth structure [26–28]. However, only few addressed the effect of acute or chronic exposure of the leaked bleaching agents to oral mucosal keratinocytes. Although 3% H_2_O_2_ was most commonly used for tooth bleaching, the actual exposure dose of to the basal cell layer of oral mucosa that regenerate the mucosal barrier was affected by multiple factors. At one end, the peroxide releases into saliva from home bleaching systems require to diffuse through the carrier while being diluted and degraded by the saliva. It has been reported that gel formulation significantly reduced the peroxide concentration in the saliva more than that of the liquid form [27]. The leaked peroxide was rapidly degraded by the saliva to 52 and 24% of the original concentrations at 2 and 6 h after exposure, respectively [29]. The remaining salivary H_2_O_2_ needs to penetrate deep into the basal layer and subgingival tissues through epithelium barrier to exert pathological effects [30]. According to EU recommend oral hygiene products, the tissue exposure of H_2_O_2_ concentration should be limited to 0.1% or 29.4 mM. In this study, the direct exposure at dose range of 0.01 mM to 100 mM was applied using primary cultured gingival oral kerayinocyte as the model. The pathological effects of both short (1 h) and long (8 h) exposures were analyzed according to the common clinical practices_._

The safety of H_2_O_2_ tooth bleaching is still controversial: its genotoxicity and carcinogenicity are under active discussion [9, 10]. Diaz-Llera et al. showed that 0.34–1.35 μM of H_2_O_2_ induced hypoxanthine guanine phosphoribosyltransferase (HPRT) mutation both in vitro and in vivo [31]. High-dose H_2_O_2_ was reported to be mildly carcinogenic for the duodenum of catalase-deficient mice [9]. In another report, 1% H_2_O_2_ (~ 0.3 M) in drinking water induced forestomach tumors in rats [10]. These reports showed that exposure to high-dose H_2_O_2_ for a sustained period induces oxidative stress that leads to DNA damage in mammalian cells.

In this study, we used primary cultured NHOKs isolated from the basal layer of oral gingival epithelium that is closely related to the most susceptible cell types in clinical practice of tooth bleaching (gingival mucosa). The primary cultured NHOKs presented similar properties of basal layer keratinocytes that maintained certain replication and differentiation potential suitable for study the peroxide induced DNA damage and subsequent pathogenic signaling. Unlike nDNA, mtDNA lacks histone protections and sophisticated DNA repair mechanisms to shelter it from oxidative attacks [32, 33]. Free radical attacks normally cause mtDNA mutation, deletion, or other types of damage and can be preserved in the cells in heteroplasmic format. One common type of mtDNA damage is the 4977-bp deletion (*nt8469–13,447*) that is related to aging [34, 35] and to different diseases, such as Kearns-Sayre syndrome [36]. A comet assay confirmed that H_2_O_2_ induced nDNA damage. In the tested dose range of 0.01 to 200 mM, we found that the number of nDNA single strand breaks was dose-dependent. Interestingly, only the difference in nDNA damage between 1 and 8 h of exposure to each dose of H_2_O_2_ was significant. However, we found no plateau dose in the treatment range.

The comet assay showed only nDNA damage but not mtDNA damage [37]. We thus used qPCR to quantify the ratio of mtDNA damage. For the control NHOKs, mtDNA^4977^ deletion showed only 0.81% baseline deletion in vitro. During the first hour of exposure to H_2_O_2_, mtDNA^4977^ deletion dose-dependently increased. Despite a significant drop in the mtDNA deletion ratio at a dose of 100 mM H_2_O_2_, we suspect that it can be attributed to direct massive cellular damage, which was supported by the MTT assay. After the NHOKs had been exposed to H_2_O_2_ for 8 h, all groups but the 100-mM treatment group were restored to their approximate baseline levels. This confirmed that H_2_O_2_ can be genotoxic to both nDNA and mtDNA in NHOKs. Moreover, in keratinocytes treated with < 10 mM of H_2_O_2_ for 8 h, mtDNA^4977^ deletion, but not nDNA, returned to normal. This is consistent with Ballinger et al., who used two treatment doses (0.1 and 0.5 mM) for 1 h [32].

Croteau and Bohr [38] reported that mitochondria are more efficient at DNA repair than nDNA, and that mitochondria are able to repair 65% of the lesions within 4 h. However, nDNA has a more sophisticated defense and repair mechanism than does mtDNA because it includes histone protection and nucleotide excision repair. Both nDNA and mtDNA damage contribute to carcinogenesis [39]. Therefore, the genotoxicity of both mtDNA and nDNA should be a concern for people who use at-home-bleaching over the long term.

Unlike nDNA damage, mtDNA damage can be preserved in a heteroplasmic state, thus allowing cells to survive and proliferate when the damage is repairable [40]. Both normal and damaged mtDNA copies can be amplified and passage in the organelles. A large-scale deletion would affect the supply of essential proteins in the electron transport chain, thus interfering with the normal function of mitochondria; this can be considered a loss of ΔΨ_m_ [41]. In the present study, ΔΨ_m_ was low at 8 h, as well as it was after 1 h, which was consistent with the treatment-time-associated alterations in the ratio of mtDNA^4977^ deletion. A dramatic (~ 407-fold) increase in ΔΨ_m_ loss occurred within 1 h after NHOKs had been treated with doses < 1 mM. For longer exposure (8 h), the degree of ΔΨ_m_ loss was attenuated to between 10- and 30-fold, and it occurred with doses as low as 0.01 mM. Thus, mtDNA^4977^ deletion seemed to be more sensitive to oxidative attacks than was ΔΨ_m_ to the dose and duration of exposure to H_2_O_2_, which is conceivable because replacing a protein complex in damaged DNA takes time through transcription and translation. The same reason applied for the observation that after 8 h, ΔΨ_m_ loss occurred even at doses as low as 0.01 mM, while the ratio of mtDNA^4977^ deletion had already been restored at doses < 100 mM.

Oxidative attacks induce various types of cell death: apoptosis, autophagy, and necrosis [42–44]. These cell death mechanisms are in fact not completely disadvantageous: they prevent severely damaged cells from contributing to the development of cancer. After 1 h of exposure to H_2_O_2_, the number of apoptotic cells dose-dependently increased, and the apoptotic fraction dropped significantly at doses > 200 mM. The number of necrotic cells increased at doses > 5 mM. At the highest dose (200 mM), a large number of NHOKs became necrotic instead of apoptotic, as evidenced by a large PI-positive population. After 8 h of exposure, apoptosis was also dose-dependent, but the apoptotic cell population rapidly fell when treatment doses were > 50 mM. The necrotic population grew as the apoptotic population declined.

At lower levels of oxidative attack, pro-apoptotic Bcl-2 family proteins increase mitochondrial membrane permeability and stimulate voltage-dependent anion channel (VDAC) activity which reduces H^+^ and ΔΨ_m_ in the inner membrane, as shown in our ΔΨ_m_ assay. When the matrix expands to rupture the outer membrane, cytochrome *c* and apoptosis-inducing factor are released to the cytoplasm to induce cell apoptosis [45–47]. The observed timing and dose response in our experiments support this model. In addition, a drastic decline of cell viability at high doses of H_2_O_2_ should be a primary consequence of direct chemically induced necrosis, but apoptosis was activated at low doses of H_2_O_2_.

In addition to DNA repair and various types of cell death, activation of antioxidant molecular mechanisms is important for cellular defense against at-home-bleaching-induced health hazards. It is known that catalase and GSH are essential for the cellular antioxidant defense system, and that they detoxify H_2_O_2_-induced damage in vivo [48]. GSH is one of the most important nonenzymatic antioxidants that exist in large amounts within cells, including in mitochondria [22]. Thus, measuring GSH content in response to different doses of H_2_O_2_ should provide important mechanistic insights. After the first hour of treatment, GSH levels were higher than at baseline when H_2_O_2_ doses were between 0.01 mM and 5 mM. This was followed by a dose-dependent decline from 86 to 16% of baseline in the dose range of 10 mM to 200 mM, which implies that at lower doses, a protective increase in GSH was activated. At a high dose, however, the endogenous GSH was rapidly consumed to protect cells against oxidative attacks. After the NHOKs had been exposed to H_2_O_2_ for 8 h, excess GSH was detected when the dose was < 0.1 mM, but the levels were significantly lower than they had been. At higher doses, dose-dependent decreases in GSH were detected, but they were consistently lower than they had been at the same doses. These findings implied that a negative balance between GSH generation and consumption occurred during continuous exposure to H_2_O_2_.

## Conclusion

Our study showed the importance for keratinocyte damage affected by the dose and the duration of H_2_O_2_ exposure in at-home-bleaching. A treatment dose ≥100 mM directly causes severe cytotoxicity within as little as 15 min of exposure. According to other reports [49], saliva can readily convert high doses (~ 1.6 M) of H_2_O_2_ to as little as 0.029 mM in 20 min. At such doses, mtDNA^4977^ deletion is expected to slightly increase and the apoptotic population to double during the first hour of exposure. The deletion ratio will be restored after 8 h of exposure, but apoptotic cells will quadruple in number. However, GSH at such a high treatment dose was significantly elevated and fewer nDNA breaks were detected. To verify the biological safety and feasibility of H_2_O_2_ tooth bleaching for long-term use, additional in vivo studies in which the local tissue environment and circulation factors are controlled must be conducted.

## Abbreviations

GSH: glutathione
GSSG: oxidized glutathione
H_2_O_2_: Hydrogen peroxide
HPRT: hypoxanthine guanine phosphoribosyltransferase
JC-1: 5, 5′, 6, 6′-tetrachloro-1, 1′, 3, 3′-tetraethylbenzimidazolcarbocyanine iodide
KGM: Keratinocyte-SFM medium
mtDNA: mitochondrial DNA
MTT: 1-(4, 5-dimethylthiazol-2-yl)-3, 5-diphenylformazan
nDNA: nuclear DNA
NHOKs: normal human oral keratinocytes
PBS: phosphate-buffered saline
PI: pyridium iodide
PSF: penicillin streptomycin and fungizone
qPCR: real-time quantitative polymerase chain reaction
SCGE: single cell gel electrophoresis
SSB: single strand break
TI: Tail intensity
TL: Tail length
TM: tail moment
VDAC: voltage-dependent anion channel
ΔΨ_m_: mitochondrial Membrane Potential
